# Visit-to-visit fasting plasma glucose variability is an important risk factor for long-term changes in left cardiac structure and function in patients with type 2 diabetes

**DOI:** 10.1186/s12933-019-0854-9

**Published:** 2019-04-16

**Authors:** Xixiang Tang, Junlin Zhong, Hui Zhang, Yanting Luo, Xing Liu, Long Peng, Yanling Zhang, Xiaoxian Qian, Boxiong Jiang, Jinlai Liu, Suhua Li, Yanming Chen

**Affiliations:** 1 Department of Endocrinology & Metabosim, Guangdong Provincial Key Laboratory of Diabetology, The Third Affiliated Hospital, Sun Yat-sen University, Guangzhou, 510630 China; 2 Advanced Medical Center, The Third Affiliated Hospital, Sun Yat-sen University, Guangzhou, 510630 China; 3 Department of Ultrasonography, The Third Affiliated Hospital, Sun Yat-sen University, Guangzhou, 510630 China; 4 Department of Cardiology, The Third Affiliated Hospital, Sun Yat-sen University, Guangzhou, 510630 China

**Keywords:** Type 2 diabetes, Fasting plasma glucose, Glucose variability, Echocardiography, Cardiac structure

## Abstract

**Background:**

To investigate the effect of visit-to-visit fasting plasma glucose (FPG) variability on the left cardiac structure and function in patients with type 2 diabetes mellitus (T2DM).

**Methods:**

In this prospective cohort study, 455 T2DM patients were included and follow-up for a median of 4.7 years. FPG measured on every hospital visit was collected. FPG variability was calculated by its coefficient of variation (CV-FPG). Left cardiac structure and function were assessed using echocardiography at baseline and after follow-up. Multivariable linear regression analyses were used to estimate the effect of FPG variability on the annualized changes in left cardiac structure and function. Subgroup analysis stratified by mean HbA1c levels (< 7% and ≥ 7%) were also performed.

**Result:**

In multivariable regression analyses, CV-FPG was independently associated with the annualized changes in left ventricle (β = 0.137; *P* = 0.031), interventricular septum (β = 0.215; *P* = 0.001), left ventricular posterior wall thickness (β = 0.129; *P* = 0.048), left ventricular mass index (β = 0.227; *P* < 0.001), and left ventricular ejection fraction (β = − 0.132; *P* = 0.030). After additionally stratified by mean HbA1c levels, CV-FPG was still independently associated with the annualized changes in the above parameters in patients with HbA1c ≥ 7%, while not in patients with HbA1c < 7%.

**Conclusions:**

Visit-to-visit variability in FPG could be a novel risk factor for the long-term adverse changes in left cardiac structure and systolic function in patients with type 2 diabetes.

*Trial registration* ClinicalTrials.gov (NCT02587741), October 27, 2015, retrospectively registered.

**Electronic supplementary material:**

The online version of this article (10.1186/s12933-019-0854-9) contains supplementary material, which is available to authorized users.

## Background

Type 2 diabetes mellitus (T2DM) increases the risk for cardiovascular diseases (CVDs), including heart failure and cardiac dysfunction, which is the leading cause of diabetes-related morbidity and mortality [1]. T2DM can contribute to the adverse changes in cardiac structure and function [2]. Cumulative exposure to hyperglycemia and higher insulin resistance are risk factors for adverse left ventricle (LV) remodeling and subclinical LV dysfunction [3, 4].

Accumulate evidences shown that glycemic variability, irrespective of the magnitude of hyperglycemia, was an independent risk factor for CVDs [5], ischemic stroke [6], cardiovascular mortality [7] and all-cause mortality [8–11] in T2DM patients. However, no study has examined the effects of visit-to-visit glycemic variation, determined by coefficient of variation of fasting plasma glucose (CV-FPG), on the left cardiac structure and function.

Therefore, the objective of this study was to examine the association of visit-to-visit FPG variability with long-term changes in left cardiac structure and function among patients with T2DM, irrespective of HbA1c and other conventional risk factors.

## Methods

### Study population

This is a prospective observational cohort study conducted in our hospital between January 2013 and June 2018. A cohort of T2DM patients who admitted in hospital for glycemic control were enrolled from January 2013 to December 2014. Participants finished the final visit from January 2018 to June 2018. T2DM was diagnosed according to the 1999 criteria of the World Health Organization (WHO) [12]. Patients with overt heart failure with a New York Heart Association (NYHA) functional classification of III and IV, left ventricular ejection fraction (LVEF) less than 50%, history of primary cardiomyopathy disease, severe valvulopathy and chronic atrial fibrillation were excluded. Ethics approval was obtained from the third affiliated hospital of Sun Yat-sen University Network Ethics Committee. Informed consent was obtained from all participants.

### Data collection and follow-up

Demographic and clinical characteristics, including age, gender, body height, weight, systolic blood pressure (SBP), diastolic blood pressure (DBP), lifestyles (smoking status and alcohol consumption), comorbidity, duration of diabetes, diabetes therapy (oral hypoglycemic drug, insulin injection, or both) and medications were obtained via paper-based case report forms. The European Society of Hypertension (ESH) and of the European Society of Cardiology (ESC) defined categorical hypertension as a blood pressure ≥ 140 mmHg systolic or ≥ 90 mmHg diastolic or current use of antihypertensive medication [13]. Urinary albumin was measured in three consecutive urine collections using a turbidimetric immunoassay and expressed as the urinary microalbumin/creatinine ratio (UACR). For biochemical analysis, blood samples were drawn after 8-h overnight fasting and examined by HITACHI 7180 automatic-analyzer. The biochemical parameters included blood urea nitrogen, creatinine, uric acid, triglycerides, total cholesterol, high density lipoprotein cholesterol (HDL-C), and low density lipoprotein cholesterol (LDL-C). Dyslipidemia was defined as total cholesterol ≥ 6.22 mmol/L (≥ 240 mg/dL), and/or serum triglyceride levels ≥ 1.7 mmol/L (≥ 150 mg/dL), and/or LDL-C ≥ 4.14 mmol/L (≥ 160 mg/dL), and/or HDL-C < 1.04 mmol/L (< 40 mg/dL), and/or use of lipid-lowering medications [14]. Estimated glomerular filtration rate (eGFR) was calculated using the Modified Diet in Renal Disease (MDRD) formula (National Kidney Foundation Calculator for Healthcare Professionals). Fasting and postprandial C-peptide/insulin were determined by radioimmunoassay. The homoeostasis model assessment of insulin resistance (HOMA-IR) was calculated according to the formula: Fasting insulin*FPG/22.5 [15]. Clinical following-up was arranged every 1–3 months (depending on the glycemic control status). Anthropometric information, medications, self-monitoring of blood glucose, hypoglycemia events and adverse events were recorded at every follow-up visit. FPG and 2-h postprandial blood glucose (2 h-PBG) were assayed every 1–3 months to assess the glycemic control status by the glucose oxidase method. HbA1c was measured quarterly by D-10 hemoglobin Testing Program (Bio-Rad) with high performance liquid chromatography (HPLC). Comprehensive standardized echocardiography was performed to assess the cardiac structure and function at the inception and final visit of the study. The anti-hyperglycemic therapy and the lifestyle intervention have been evaluated by specialist physicians based on the glycemic control status during the following-up visits.

### Assessment of FPG variability

Participants with at least ten FPG determinations measurements during the study were finally included for glycemic variation calculation. For each participants, the intra-personal mean and standard deviation (SD) of all recorded FPG measurements were calculated. The CV-FPG was the ratio of the SD over the mean FPG, and the CV-FPG was considered as measure of FPG variability. Considering that the frequency of individual visits could influence the calculation, the CV-FPG was further adjusted by dividing by the square root of the ratio of total visits divided by total visits minus 1 [6, 9, 16]. The CV of 2 h-PBG and HbA1c were also calculated respectively.

### Echocardiography examination

Transthoracic echocardiography (IE33 echocardiography system) were performed on all participants according to the recommendations of the American Society of Echocardiography [17] by two trained, registered cardiac sonographers. The echocardiographic methods were designed to be as similar as possible for each individual at the baseline and follow-up time-points, with minor changes in measurements. Left atrium (LA) size was measured at the end of LV systole, when the LA chamber was at its greatest dimension. The left ventricular internal end-diastole dimension (LVDd), interventricular septum (IVS) and left ventricular posterior wall thicknesses (LVPW) were measured at the end of LV diastole, acquired in the parasternal long-axis view at the level of the mitral valve leaflet tips. LV mass index (LVMI) was measured from M-mode LV mass measurement using the Derveux corrected formula and indexed to body surface area; LV systolic function was assessed by calculating LVEF with the modified Simpson biplane method. By the pulsed wave Doppler in the apical 4-chamber view, LV inflow was obtained (peak early (E) and late (A) diastolic velocities and E/A ratio were obtained). Peak early diastolic mitral annular velocity (e′) was measured at the junction of the interventricular septum with the mitral annulus in the apical 4-chamber view by pulsed-wave tissue Doppler imaging (TDI), and the septal E/e′ ratio was calculated.

### Statistical analysis

Database management and statistical analysis was performed by using SPSS 22.0 for Windows (SPPS Inc, Chicago, IL, USA). Descriptive statistics are presented as mean ± standard deviation for continuous variables or as numbers and percentages for categorical variables. All patients were grouped into quartiles of CV-FPG (Q1 = the first quartile; Q2 = the second quartile; Q3 = the third quartile; Q4 = the fourth quartile). Continuous variables were compared by ANOVA test, while categorical variables were compared using the Pearson Chi square test. Pearson Chi square tests for trend and Spearman rank correlation tests were used to test for a relationship between the parameters of cardiac structure and function and values of CV-FPG. Univariate linear regression was performed to assess the non-adjusted relationships between FPG variability and annualized change in cardiac structure and function. After that, two multivariate linear regression models were performed to adjust the confounding factors. The first model was adjusted for age, gender, body mass index (BMI), diabetes duration, cardiovascular disease, hypertension, TG, HDL-C, LDL-C, smoking status, alcohol intake, medications, HbA1c, CV-HbA1c, PBG, CV-PBG, hypoglycemia rate and the number of FPG measurements. The second model was additionally adjusted for the corresponding mean FPG to investigate whether the association was independent of mean FPG. To determine whether the glycemic control status affect the relationship between FPG variability and cardiac structure and function, subgroup analysis was performed based on mean HbA1c levels (< 7.0% and ≥ 7.0%). A 2-tailed P value < 0.05 was considered to indicate statistical significance.

## Results

### Participants characteristics

**Fig. 1:**
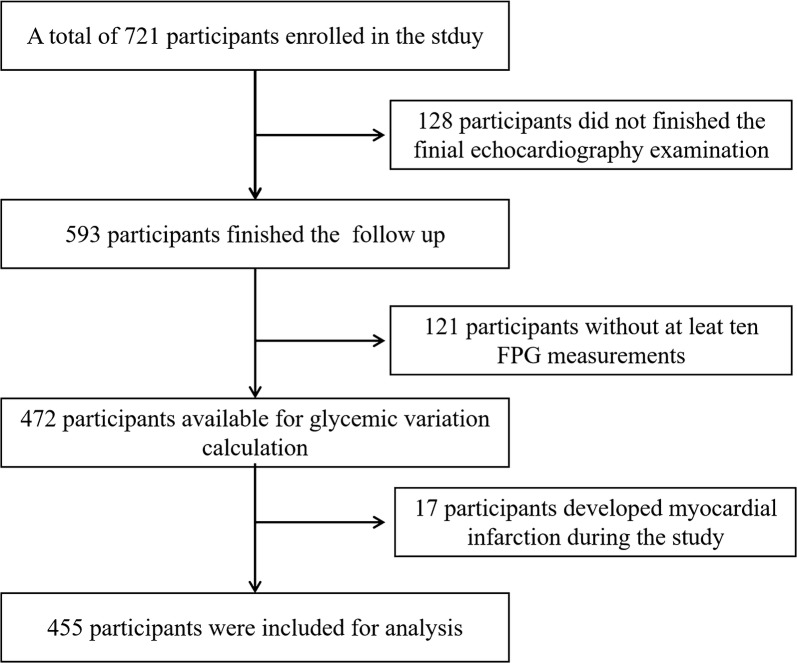
Flowchat of the present study

From January 2013 to December 2014, 721 (420 men and 301 women; aged 30–80 years) patients agreed to enroll in the study. During a median of 4.7 years of follow-up, a total of 128 participants who did not finish the finial echocardiography examination were excluded from the study. Another 121 participants with less than ten FPG measurements during the study were also excluded. In addition, we also excluded 17 individuals who developed myocardial infarction during follow-up period. Finally, the remaining 455 participants (218 men and 235 women; mean age was 60.11 ± 12.73 years) were eligible for analysis (Fig. 1). The median of diabetes duration of the study cohort is 6.3 (2.3-10.4) years, with a mean HbA1c of 7.01 ± 1.05%. The mean FPG was 7.24 ± 1.54 mmol/L, and the CV of which is 39.01 ± 14.96%. Table 1 showed the baseline sociodemographic and clinical factors in subjects grouped according to quartiles of CV-FPG. The values of CV-FPG ranged from 6.26 to 26.56% in Q1 subjects, 26.56 to 34.99% in Q2 subjects, 34.99 to 44.89% in Q3 subjects, and 44.89 to 95.40% in Q4 subjects. The diabetic duration, mean HbA1c, CV-HbA1c, CV-PBG, proportion of insulin injection, and proportion of calcium channel blocker intake varied among groups (*P* < 0.01). However, no significant differences were observed in the other baseline profiles among subjects with different CV-FPG quartiles.

**Table 1:** Baseline characteristics of patients grouped by quartile of CV-FPG levels

Variables (% or mean ± SD)	Quartile of CV-FPG				P-value
	1	2	3	4	
Quartile range	≤ 26.56	26.56–34.99	34.99–44.89	> 44.89	
n	114	114	114	113	
Male, n (%)	65 (57.01)	44 (38.60)	58 (50.88)	51 (45.13)	0.037
Age, years	58.3 ± 13.3	60.9 ± 12.0	61.0 ± 12.0	60.2 ± 12.9	0.347
BMI, kg/m^2^	22.63 ± 5.20	22.08 ± 4.45	22.07 ± 5.22	22.10 ± 4.83	0.793
Baseline SBP, mmHg	135.5 ± 12.8	134.1 ± 16.4	133.4 ± 16.8	133.8 ± 20.5	0.798
Baseline DBP, mmHg	75.4 ± 12.8	75.2 ± 15.4	75.1 ± 13.6	73.4 ± 14.3	0.698
HR, bpm	75.2 ± 12.8	75.3 ± 14.9	74.4 ± 14.2	81.5 ± 15.1	< 0.001
Follow up time, years	4.3 (3.8–5.6)	4.5 (3.8–6.8)	4.8 (4.0–6.1)	5.0 (4.0–6.1)	0.100
Blood biochemical indices					
Cr, μmol/L	89.06 ± 40.85	107.72 ± 77.90	104.89 ± 78.12	98.81 ± 47.97	0.125
eGFR, mL/min/1.73 m^2^	78.31 ± 31.23	72.65 ± 21.99	74.38 ± 27.86	73.00 ± 34.00	0.460
BUN, μmol/L	6.80 ± 2.32	7.59 ± 4.89	7.86 ± 4.33	7.16 ± 2.85	0.159
UA, μmol/L	361.61 ± 115.21	388.81 ± 177.93	376.29 ± 117.47	384.97 ± 115.60	0.302
TC, mmol/L	4.50 ± 1.08	4.80 ± 1.34	4.80 ± 1.14	4.92 ± 1.38	0.071
TG, mmol/L	1.78 (1.30–3.84)	3.03 (1.65–4.13)	2.09 (1.28–3.25)	2.78 (1.56–4.54)	0.092
HDL-C, mmol/L	1.25 ± 0.35	1.23 ± 0.31	1.24 ± 0.31	1.23 ± 0.30	0.924
LDL-C, mmol/L	2.76 ± 0.96	3.05 ± 0.85	2.91 ± 0.82	3.00 ± 0.92	0.068
Lifestyles, n (%)					
Smoking, n (%)	25 (21.93)	32 (28.07)	38 (33.33)	34 (30.09)	0.275
Alcohol, n (%)	25 (21.93)	32 (28.07)	34 (29.82)	26 (23.01)	0.453
Diabetes-related variables					
Diabetes duration, years	3.35 (1.40–8.00)	6.80 (3.40–10.15)	8.75 (3.08–14.25)	5.60 (2.00–10.90)	< 0.001
Mean of FPG, mmol/L	7.26 ± 1.64	7.47 ± 1.41	7.31 ± 1.58	6.94 ± 1.50	0.073
CV-FPG, %	20.77 ± 4.73	30.94 ± 2.53	39.56 ± 2.99	56.77 ± 9.31	< 0.001
Minimum FPG, mmol/L	4.1	5.25	4.03	3.5	–
Maximum FPG, mmol/L	11.62	11.91	10.96	11.13	–
Mean of PBG, mmol/L	8.86 ± 1.55	8.97 ± 1.41	8.88 ± 1.49	8.51 ± 1.40	0.091
CV-PBG, %	21.29 ± 6.04	23.67 ± 5.51	29.33 ± 7.57	37.66 ± 9.84	< 0.001
Mean of HbA1c, %	6.76 ± 0.95	6.98 ± 0.96	6.89 ± 0.91	7.41 ± 1.23	< 0.001
CV-HbA1c, %	19.62 ± 6.67	21.29 ± 7.72	22.13 ± 10.29	23.43 ± 6.84	0.004
FCP, nmol/L	1.72 ± 1.10	1.95 ± 1.33	1.73 ± 0.90	1.85 ± 1.26	0.373
2 h-PCP, nmol/L	4.96 ± 2.64	4.99 ± 2.27	4.55 ± 2.23	4.66 ± 2.44	0.414
Fasting insulin, mu/L	74.27 ± 44.86	80.09 ± 47.57	66.80 ± 39.64	77.40 ± 48.46	0.140
2 h-insulin, mu/L	228.47 ± 138.98	241.32 ± 159.14	208.21 ± 142.37	222.65 ± 149.47	0.399
HOMA-IR	18.92 (11.78–33.42)	21.89 (13.00–36.11)	18.10 (11.48–26.26)	18.58 (11.57–31.63)	0.078
UACR, mg/g	4.13 (2.53–8.80)	4.80 (2.53–11.17)	4.73 (2.60–7.67)	4.13 (2.79–8.50)	0.721
Hypoglycemia rates, %	33 (28.9)	29 (25.4)	37 (32.5)	29 (25.7)	0.606
Medications, n (%)					
Insulin, n (%)	53 (46.5)	62 (54.4)	69 (60.5)	77 (68.1)	0.008
OADs, n (%)	91 (79.8)	89 (78.1)	85 (74.6)	80 (70.8)	0.397
Sulfonylureas, n (%)	22 (19.30)	22 (19.30)	28 (24.56)	20 (17.70)	0.594
Glinides, n (%)	7 (6.14)	7 (6.14)	2 (1.75)	2 (1.77)	0.125
Metformin, n (%)	56 (49.12)	54 (47.37)	56 (49.12)	43 (38.05)	0.278
Thiazolidinediones, n (%)	0	0	0	0	–
Glucosidase inhibitor, n (%)	42 (36.84)	39 (34.21)	37 (32.46)	38 (33.63)	0.914
DPP-4 inhibitors/GLP-1 receptor agonists, n (%)	10 (8.77)	13 (11.40)	11(9.65)	10 (8.85)	0.900
Anti-platelet, n (%)	55 (48.25)	57 (50.00)	58 (50.88)	60 (53.10)	0.907
Statin, n (%)	60 (52.63)	58 (50.88)	69 (60.53)	59 (52.21)	0.452
ACEI/ARB, n (%)	51 (44.74)	42 (36.84)	53 (46.49)	55 (48.67)	0.299
β_blocker, n (%)	47 (41.23)	44 (38.60)	35 (30.70)	44 (38.94)	0.379
CCB, n (%)	47 (41.23)	61 (53.51)	46 (40.35)	38 (33.63)	0.022
Diuretic, n (%)	29 (25.44)	32 (28.07)	36 (31.58)	29 (25.66)	0.708
Comorbidity, n (%)					
CAD, n (%)	37 (32.46)	38 (33.33)	38 (33.33)	37 (32.74)	0.999
Hyperlipidemia, n (%)	52 (45.61)	54 (47.37)	55 (48.25)	52 (46.02)	0.977
Hypertension, n (%)	52 (45.61)	55 (48.25)	48 (42.11)	63 (55.75)	0.206

Data are mean ± SD, median (25th to 75th percentile) or n (%)

SBP, systolic blood pressure; DBP, diastolic blood pressure; FPG, fasting plasma glucose; Cr, plasma creatinine; eGFR, estimated glomerular filtration rate; BUN, blood urea nitrogen; UA, uric acid; TC, total cholesterol; TG, triglycerides; HDL-C, high density lipoprotein cholesterol; LDL-C, low density lipoprotein cholesterol; PBG, postprandial blood glucose; FCP, Fasting C-peptide; 2 h-PCP, 2 h-postprandial C-peptide; HOMA-IR, homeostatic model assessment for Insulin Resistance; UACR, urinary microalbumin/creatinine ratio; OADs, oral antidiabetic drugs; DPP-4, Dipeptidyl peptidase-4; GLP-1, Glucagon-like peptide-1; ACEI/ARB, angiotensin converting enzyme inhibitor/angiotensin receptor blocker; CCB, calcium channel blockers; CAD, cardiac artery disease

### Echocardiographic parameters stratified by CV-FPG quartiles

The baseline echocardiographic parameters of the whole cohort manifested as normal left cardiac structure and systolic function, along with mild diastolic dysfunction. Over a median of 4.7-year follow-up, adverse changes could be observed in most of the parameters of left cardiac structure and function, as showed in Additional file 1: Table S1.

**Fig. 2:**
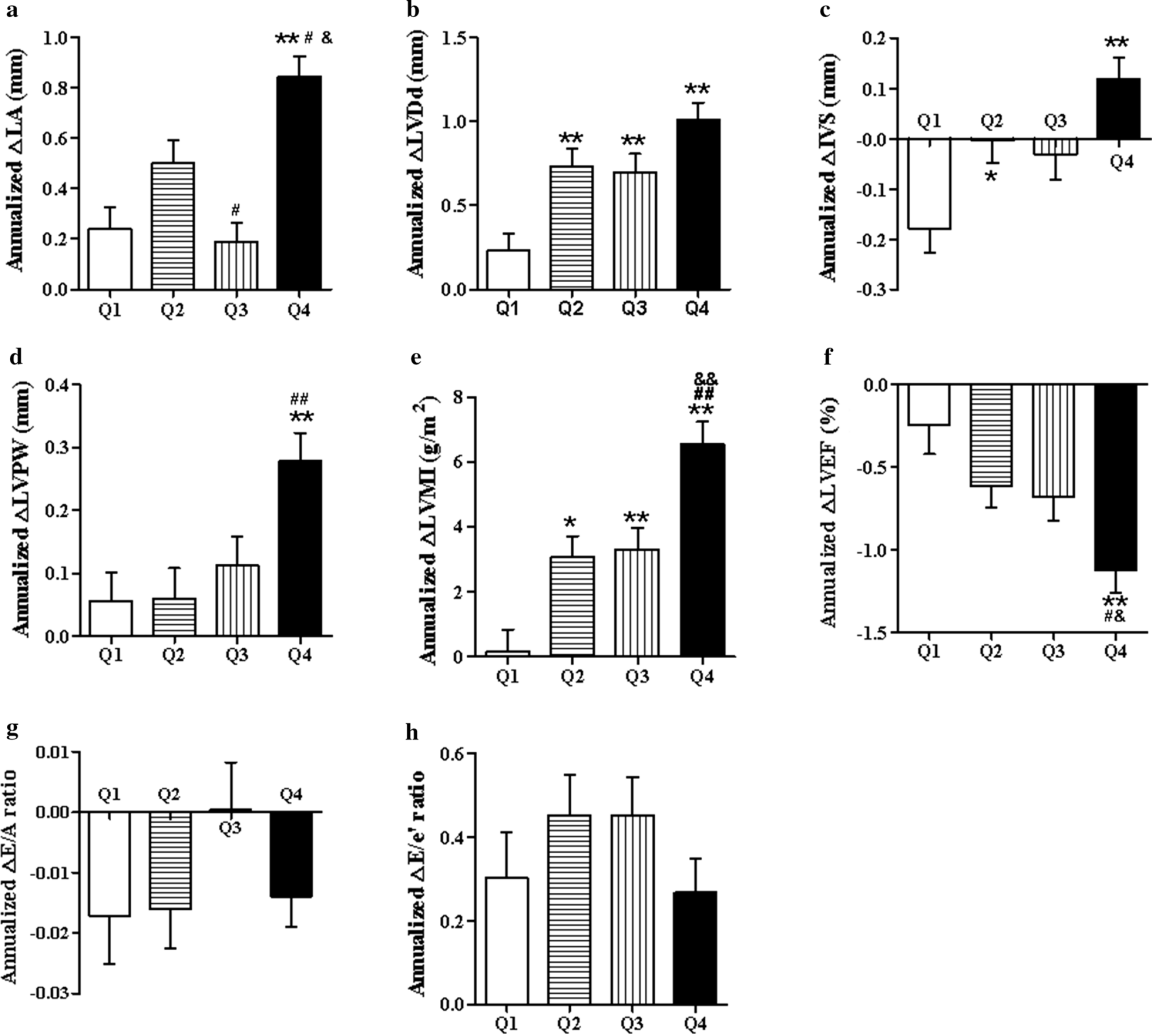
Annualized change in left cardiac structural and functional parameters of patients grouped by quartile of CV-FPG levels. **a** Anualized change in LA (**a**), LVDd (**b**), IVS (**c**), LVPW (**d**), LVMI (**e**), LVEF (**f**), E/A ratio (**g**) and E/e′ ratio (**h**) among groups divided by quartile of CV-FPG levels. Data are shown as mean ± SD. *P < 0.05, **P < 0.01 versus the Q1 group; ^#^*P* < 0.05, ^##^*P* < 0.01 versus the Q2 group; ^&^*P* < 0.05, ^&&^*P* < 0.01 versus the Q3 group by one-way ANOVA. LA, left atrium; LVDd, left ventricular internal end-diastole dimension; IVS, interventricular septum; LVPW, left ventricular posterior wall thicknesses; LVMI, left ventricular mass index; LVEF, left ventricular ejection fraction; Q1, the first quartile; Q2, the second quartile; Q3, the third quartile; Q4, the fourth quartile

After further stratified by quartiles of CV-FPG, measures of left cardiac structure and function were showed in Table 2. The baseline LVDd, LVMI, LVEF and A velocity varied among subjects with different CV-FPG quartiles, while no differences were observed in LA, IVS, LVPW, E/A ratio and E/e’ ratio. In order to reduce the impact of baseline inconsistency, we therefore use annualized changes of echocardiographic parameters to evaluate the effect of CV-FPG on left cardiac structure and function, as shown in Fig. 2. After a median of 4.7-year follow-up, elevating trends from Q1 to Q4 were showed in the annualized change of LA (*P* < 0.001), LVDd (*P* < 0.001), IVS (*P* < 0.001), LVPW (*P* = 0.002) and LVMI (*P* < 0.001), while declined trend was observed in the annualized change of LVEF. However, no significant differences were detected in annualized changes of parameters of LV diastolic function (E/A ratio and E/e′) among participants stratified by CV-FPG quartiles (*P* > 0.05).

**Table 2:** The baseline and follow-up left cardiac structural and functional parameters of patients grouped by quartile of CV-FPG levels

Variables	Baseline					After				
	1	2	3	4	*P*-value	1	2	3	4	*P*-value
Left cardiac structure										
LA, mm	32.20 ± 6.00	31.25 ± 5.09	31.87 ± 4.89	30.54 ± 4.88	0.086	33.26 ± 5.61	33.54 ± 5.00	32.82 ± 4.85	34.49 ± 5.01	0.095
LVDd, mm	47.40 ± 4.63	44.75 ± 4.11	44.54 ± 4.32	44.65 ± 3.85	< 0.001	48.51 ± 4.52	48.12 ± 4.25	47.63 ± 4.70	49.44 ± 3.87	0.015
IVS, mm	12.87 ± 2.06	12.60 ± 2.04	12.63 ± 1.85	12.44 ± 1.75	0.418	11.90 ± 2.19	12.53 ± 1.81	12.44 ± 2.00	13.09 ± 1.93	< 0.001
LVPW, mm	9.34 ± 1.60	9.51 ± 1.72	9.40 ± 1.69	9.60 ± 1.63	0.653	9.67 ± 1.47	9.78 ± 1.62	9.93 ± 1.76	10.93 ± 1.66	< 0.001
LVMI, g/m^2^	110.46 ± 30.64	99.01 ± 25.14	98.61 ± 25.26	99.11 ± 26.58	0.001	110.55 ± 32.16	112.73 ± 29.13	113.23 ± 34.44	130.85 ± 31.34	< 0.001
Left ventricular systolic function										
LVEF, %	57.06 ± 5.32	58.82 ± 5.57	58.91 ± 5.93	57.22 ± 5.19	0.011	55.75 ± 7.41	56.20 ± 6.00	55.51 ± 6.35	51.90 ± 7.37	< 0.001
Left ventricular diastolic function										
E velocity, cm/s	0.74 ± 0.19	0.77 ± 0.22	0.79 ± 0.25	0.78 ± 0.23	0.296	0.77 ± 0.18	0.80 ± 0.21	0.85 ± 0.26	0.82 ± 0.23	0.029
A velocity, cm/s	0.80 ± 0.19	0.83 ± 0.20	0.91 ± 0.33	0.82 ± 0.24	0.007	0.88 ± 0.21	0.93 ± 0.27	0.97 ± 0.32	0.92 ± 0.27	0.117
E/A ratio	0.98 ± 0.36	0.97 ± 0.33	0.95 ± 0.36	1.00 ± 0.33	0.677	0.91 ± 0.31	0.89 ± 0.26	0.93 ± 0.34	0.94 ± 0.32	0.721
Tissue Doppler e′, cm/s	0.10 ± 0.03	0.11 ± 0.03	0.11 ± 0.03	0.11 ± 0.03	0.220	0.09 ± 0.03	0.09 ± 0.02	0.09 ± 0.02	0.09 ± 0.03	0.936
E/e′ ratio	7.67 ± 3.10	7.41 ± 2.91	7.69 ± 3.36	7.89 ± 3.58	0.740	9.04 ± 3.87	9.28 ± 3.65	9.78 ± 4.21	9.25 ± 3.61	0.517

Data are mean ± SD, median (25th to 75th percentile) or n (%)

LA, left atrium; LVDd, left ventricular internal end-diastole dimension; IVS, interventricular septum; LVPW, left ventricular posterior wall thicknesses; LVMI, left ventricular mass index; LVEF, left ventricular ejection fraction

### Univariate and multivariate linear regression analysis

**Fig. 3:**
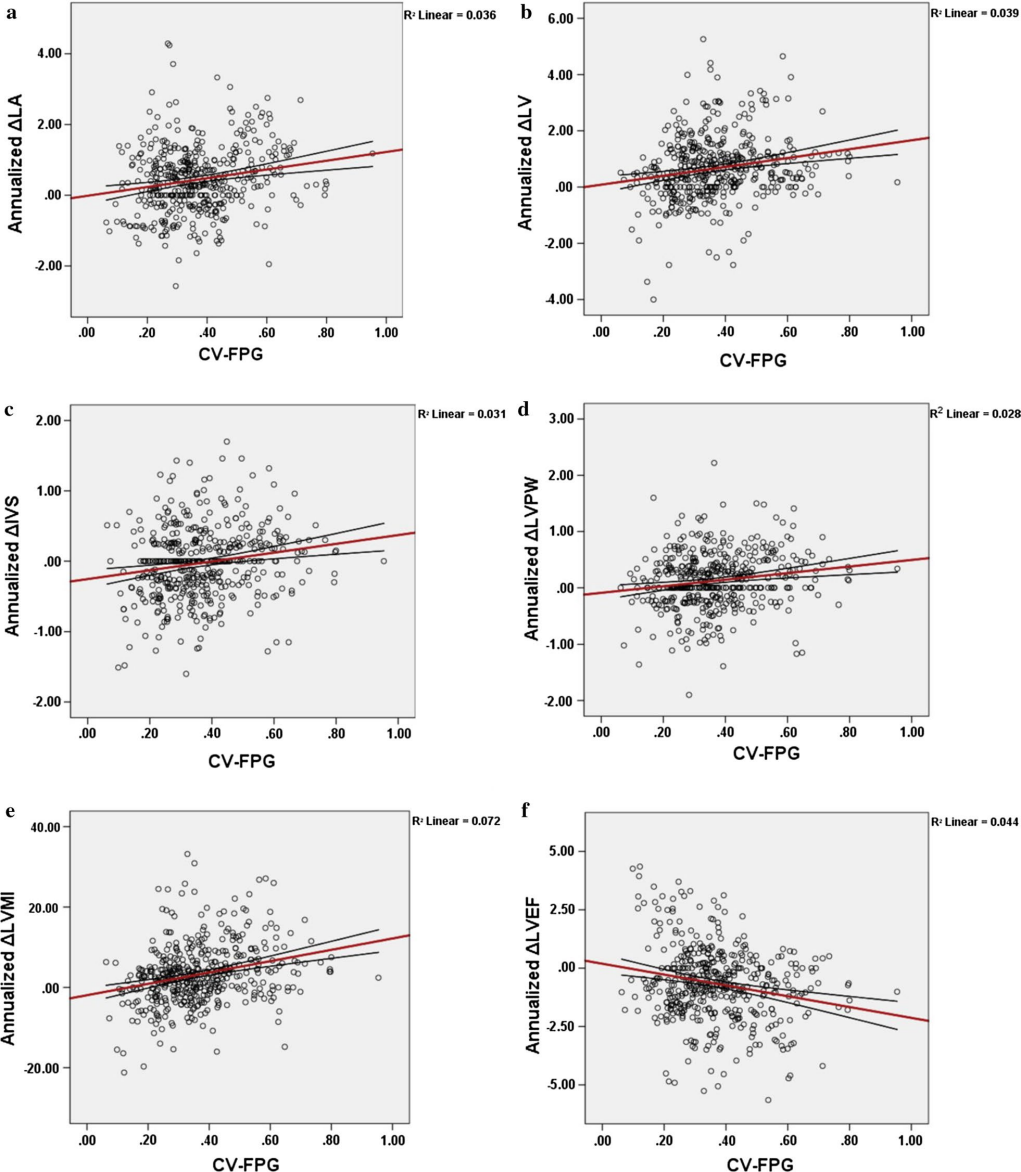
Correlation between annualized changes of left cardiac structure and function and CV-FPG.** a** Y = − 0.02 + 1.24 * X, R linear = 0.190, R^2^ linear = 0.036, P < 0.001;** b** Y = − 0.08 + 1.58 * X, R linear = 0.199, R^2^ linear = 0.039, P < 0.001; **c** Y = − 0.25 + 0.63 * X, R linear = 0.175, R^2^ linear = 0.031, P < 0.001; **d** Y = − 0.09 + 0.58 * X, R linear = 0.168, R2 linear = 0.028, P < 0.001; **e** Y = − 1.95 + 14.12 * X, R linear = 0.269, R^2^ linear = 0.072, P < 0.001; **f** Y = 0.19 − 2.32 * X, R linear = − 0.209, R^2^ linear = 0.044, P < 0.001.

Univariate linear regression analysis demonstrated that annualized changes of LA (*P* < 0.001), LVDd (*P* < 0.001), IVS (*P* < 0.001), LVPW (*P* < 0.001), LVMI (*P* < 0.001), LVEF (*P* < 0.001) were significantly related to values of CV-FPG (Fig. 3 and Table 3). When further adjusting for age, gender, BMI, diabetes duration, cardiovascular disease, hypertension, TG, HDL-C, LDL-C, smoking status, alcohol intake, medications, mean HbA1c, CV-HbA1c, PBG, CV-PBG, hypoglycemia rate and the number of FPG measurements (model 1), CV-FPG remained independently associated with annualized changes of LVDd (*P* = 0.044), IVS (*P* = 0.001), LVPW (*P* = 0.045), LVMI (*P* < 0.001), LVEF (*P* < 0.001). Further adjusting for mean FPG showed that CV-FPG was still significantly related to these cardiac parameters (LVDd, IVS, LVPW, LVMI and LVEF) (model 2). No significant relationship were found between left cardiac diastolic function parameters (E/A ratio and E/e′ ratio) and the value of CV-FPG neither in univariate or multivariate linear regression analysis (*P* > 0.05).

**Table 3:** Linear regression analysis assessing the relationships of CV-FPG with annualized left cardiac structural and functional parameters

Cardiac structural and functional parameters	Univariate		Model 1*		Model 2**	
	β (SE)	P-value	β (SE)	P-value	β (SE)	P-value
Annualized ΔLA, mm	0.190 (0.301)	< 0.001	0.043 (0.401)	0.488	0.047 (0.403)	0.448
Annualized ΔLVDd, mm	0.199 (0.366)	< 0.001	0.128 (0.501)	0.044	0.137 (0.503)	0.031
Annualized ΔIVS, mm	0.175 (0.165)	< 0.001	0.206 (0.227)	0.001	0.215 (0.228)	0.001
Annualized ΔLVPW, mm	0.168 (0.162)	< 0.001	0.136 (0.226)	0.045	0.129 (0.227)	0.048
Annualized ΔLVMI, g/m^2^	0.269 (2.378)	< 0.001	0.217 (3.201)	< 0.001	0.227 (3.209)	< 0.001
Annualized ΔLVEF, %	− 0.209 (0.511)	< 0.001	− 0.133 (0.671)	< 0.001	− 0.132 (0.672)	0.030

Δ: The change of the cardiac parameters from baseline to the final visit, Β, standardized β-estimates; SE, standard error; LA, left atrium; LVDd, left ventricular internal end-diastole dimension; IVS, interventricular septum; LVPW, left ventricular posterior wall thicknesses; LVMI, left ventricular mass index; LVEF, left ventricular ejection fraction; TG, triglycerides; HDL-C, high density lipoprotein cholesterol; LDL-C, low density lipoprotein cholesterol

*Model 1 was adjusted for age, gender, body mass index, duration of diabetes mellitus, cardiovascular disease, TG, HDL-C, LDL-C, hypertension, smoking status, alcohol intake, medications, HbA1c, CV-HbA1c, PBG, CV-PBG, hypoglycemia rate and the number of FPG measurements

**Model 2 was additionally adjusted for the corresponding FPG values

### Subgroup analysis

An reasonable HbA1c level is below or around 7% according to the American Diabetes Association (ADA) and the European Association for the Study of Diabetes (EASD) [18]. In order to reduce the influence of glucose control status, we further performed subgroup regression analysis stratified by mean HbA1c levels (< 7% vs. ≥ 7%), as shown in Table 4. In multivariate liner regression model 1 and model 2, CV-FPG was significantly related with LVDd, IVS, LVPW, LVMI and LVEF in the subgroup of HbA1c ≥ 7% (*P* < 0.05), but not in the subgroup of HbA1c < 7% (*P* > 0.05).

**Table 4:** Subgroup regression analysis stratified by the level of HbA1c

Cardiac structural and functional parameters	Univariate		Model 1*		Model 2**	
	β (SE)	P-value	β (SE)	P-value	β (SE)	P-value
Annualized ΔLA, mm						
HbA1c < 7.0	0.172 (0.433)	< 0.001	− 0.026 (0.626)	0.782	− 0.020 (0.629)	0.828
HbA1c ≥ 7.0	0.178 (0.431)	0.008	0.108 (0.541)	0.198	0.108 (0.547)	0.201
Annualized ΔLV, mm						
HbA1c < 7.0	0.207 (0.537)	0.002	0.047 (0.776)	0.614	0.056 (0.778)	0.547
HbA1c ≥ 7.0	0.171 (0.515)	0.002	0.223 (0.702)	0.014	0.234 (0.707)	0.011
Annualized ΔIVS, mm						
HbA1c < 7.0	0.122 (0.227)	0.062	0.168 (0.331)	0.080	0.177 (0.332)	0.066
HbA1c ≥ 7.0	0.184 (0.245)	0.006	0.283 (0.338)	0.002	0.294 (0.340)	0.002
Annualized ΔLVPW, mm						
HbA1c < 7.0	0.092 (0.232)	0.160	− 0.005 (0.336)	0.958	− 0.006 (0.338)	0.952
HbA1c ≥ 7.0	0.250 (0.231)	< 0.001	0.240 (0.330)	0.011	0.255 (0.331)	0.007
Annualized ΔLVMI, g/m^2^						
HbA1c < 7.0	0.239 (3.248)	< 0.001	0.117 (4.644)	0.202	0.126 (4.658)	0.172
HbA1c ≥ 7.0	0.272 (3.573)	< 0.001	0.335 (4.76)	< 0.001	0.350 (4.779)	< 0.001
Annualized ΔLVEF, %						
HbA1c < 7.0	− 0.231 (0.713)	< 0.001	− 0.107 (1.007)	0.236	− 0.103 (1.012)	0.260
HbA1c ≥ 7.0	− 0.181 (0.756)	0.007	− 0.180 (0.951)	0.043	− 0.168 (0.958)	0.047

Δ: The change of the cardiac parameters from baseline to the final visit. LA, left atrium; LVDd, left ventricular internal end-diastole dimension; IVS: interventricular septum; LVPW, left ventricular posterior wall thicknesses; LVMI, left ventricular mass index; LVEF: left ventricular ejection fraction; Β, standardized β-estimates; SE, standard error

*Model 1 was adjusted for age, gender, body mass index, duration of diabetes mellitus, cardiovascular disease, TG, HDL-C, LDL-C, hypertension, smoking status, alcohol intake, medications, HbA1c, CV-HbA1c, PBG, CV-PBG, hypoglycemia rate and the number of FPG measurements

**Model 2 was additionally adjusted for the corresponding FPG values

## Discussion

This study is the first to demonstrate that visit-to-visit FPG variability, represented by CV-FPG, was associated with the subclinical left cardiac remodeling and systolic dysfunction, independently of mean glucose control status and other conventional risk factors. Our finding suggests that visit-to-visit FPG variability can be routinely monitored, and the effect of antidiabetes therapy on glucose fluctuation should be considered in the clinical management of patients with T2DM.

Abundant of studies have showed that T2DM was related to various target organ damage and elevated risk of cardiovascular events [19–21]. In addition, previous studies suggested that T2DM was associated with minute LV systolic and diastolic dysfunction, which might further lead to the progression of diabetic cardiomyopathy [20, 22–25]. T2DM patients with suboptimal glycemic control have increased aortic stiffness and a higher prevalence of LV hypertrophy, along with lower diastolic and systolic LV longitudinal performance [26]. Subclinical LA structural and functional changes were also observed in T2DM patients with normal left atrial size [27, 28]. In consistent with previous studies, the present study indicated that preclinical left cardiac remodeling and dysfunction had develop in T2DM patients before the occurrence of clinical heart failure [4, 29–31].

Glucose variability, another factor showing glycemic control abnormality in addition to conventional risk factors, has been reported to be associated with increased risk of complications and mortality in diabetes patients [32]. Independently of mean FPG and HbA1c, previous studies showed that elevated visit-to-visit variability of FPG significantly increased the risk of CVDs and all-cause mortality in patients with T2DM [8, 9]. Glycemic variability assessed by visit-to-visit HbA1c variability was inversely related to Baroreflex sensitivity, as a sensitive indicator of T2DM patients with cardiovascular autonomic neuropathy, independently of the mean HbA1c in patients with T2DM, which has been found to be associated with cardiovascular events [33]. HbA1c variability was independently predictive of all-cause death and composite events T2DM patients with heart failure [34].The present study provided further evidence that visit-to-visit variability of FPG is independently associated with subclinical echocardiographic abnormalities of LV structure and systolic function before the occurrence of clinical cardiovascular events. In accordance with our findings, especially in patients with HbA1c ≥ 7%, the higher glycemic variability was related to the left cardiac remodeling and systolic dysfunction. An excessively fast glucose-lowering rate, which indicate high glucose fluctuation, could impair LV systolic function in patients with T2DM and CAD [35]. Furthermore, glycemic variability determined with a continuous glucose monitoring system can predict prognosis in patients with acute coronary syndrome without severe diabetes [36].

In addition, accumulated evidence demonstrated that diabetes were associated with cardiac diastolic dysfunction [37–39] and patients with abnormal E/e′ have higher mortality [40, 41]. In addition, a community-based cohort found that the presence of subclinical LV diastolic dysfunction was a predictor of the incident T2DM [42]. In the present study, the diastolic function was deteriorated in the whole cohort after a median of 4.7-year follow-up, suggesting that subclinical left ventricular diastolic dysfunction gradually developed with the extended duration of diabetes [43]. However, no difference was observed in the annualized changes of both E/A ratio and E/e′ ratio among groups with different quartile of CV-FPG, suggesting no significant effect of glycemic variability on subclinical diastolic dysfunction.

Optimized postprandial glucose control was related with improved myocardial/vascular function in well-controlled T2DM patients [44]. In the present study, there was no significant difference in mean postprandial glucose, but increased postprandial glucose fluctuation, assessed by CV-postprandial glucose, with the elevated levels of CV-FPG. However, the CV-FPG was still independently associated with the LV subclinical adverse remodeling and systolic dysfunction after adjusting the postprandial glucose excursions. Elevated insulin resistance was another risk factor for adverse LV remodeling and subclinical dysfunction [3]. However, no difference was observed in the hyperinsulin and insulin resistance among groups with different level of FPG variability in the present study.

The mechanisms by which elevated fluctuation of FPG contributes to cardiac remodeling and systolic dysfunction in patients with diabetes are yet to be elucidated. There are several potential explanations. Glycemic fluctuations have been shown to increases oxidative stress [45, 46] and inflammatory cytokines [25, 47], both of which were important pathogenic factors in the development of diabetic complications, including cardiomyopathy [48–51]. Blood glucose variability can induced oxidative stress by inhibiting AKT signalling path to aggravate cardiac tissue fibrosis [52], which contribute to the cardiac remodeling and dysfunction. Furthermore, individuals with a higher variability of FPG tend to have a higher prevalence of hypoglycemia and sympathetic dysfunction, and hypoglycemia is associated with the occurrence of atherosclerotic disease [53], which are associated with cardiac remodeling.

It is also important to evaluate the effects of different antidiabetic agents on glycemic variability to attenuate the adverse progression of cardiac structure and function. In a randomized clinical trial, a 16-week treatment of dipeptidyl peptidase 4 inhibitor, vildagliptin, but no pioglitazone, reduced glycemic variability in individuals with T2DM who was inadequately controlled with metformin monotherapy [54]. In addition, the sub-group analysis from the PROLOGUE study showed that adding dipeptidyl peptidase 4 inhibitor, sitagliptin, to conventional antidiabetic regimens for 2 years in T2DM patients attenuated the annual exacerbation in the cardiac dysfunction [55]. Further studies should be designed to investigate the impacts of different hypoglycemic therapies on glycemic variability and subclinical alteration of cardiac structure and function.

There are several limitations for our study. First, considering that FPG measurements were obtained from clinical follow-up, the frequency of the FPG measurements varied among patients. Although the effect of the frequency of FPG measurements on variability had been adjusted, the difference in intervals between FPG measurements has not been adequately addressed. Second, we did not assess the relation of diabetic complications with altered LV structure and function. Finally, the observation cohort study can only show the association but not the casualty.

## Conclusion

In summary, our study demonstrated that visit-to-visit variability of FPG in patients with T2DM was independently associated with the preclinical annualized change of left cardiac structure and systolic function. Nevertheless, more studies are needed to confirm the causal relationship between FPG variability and cardiac structure and function in the diabetic population.

## Additional file

**Additional file 1: Table S1.** Baseline and follow-up cardiac structural and functional parameters in participants.

## Abbreviations

CVD: cardiovascular diseases
DM: diabetes mellitus
T2DM: type 2 diabetes mellitus
LV: left ventricular
CV: coefficient of variation
FPG: fasting plasma glucose
WHO: World Health Organization
NYHA: New York Heart Association
LVEF: left ventricular ejection fraction
SBP: systolic blood pressure
DBP: diastolic blood pressure
UACR: urinary microalbumin/creatinine ratio
HDL-C: high density lipoprotein cholesterol
LDL-C: low density lipoprotein cholesterol
eGFR: estimated glomerular filtration rate
HOMA-IR: homoeostasis model assessment of insulin resistance
2 h-PBG: 2-hour postprandial blood glucose
HPLC: high performance liquid chromatography
SD: standard deviation
LA: left atrium
LVDd: left ventricular internal end-diastole dimension
IVS: interventricular septum
LVPW: left ventricular posterior wall thicknesses
LVMI: left ventricular mass index

## References

[1] Grundy SM, Benjamin IJ, Burke GL, Chait A, Eckel RH, Howard BV (1999). Diabetes and cardiovascular disease: a statement for healthcare professionals from the American Heart Association. Circulation.

[2] MacDonald MR, Petrie MC, Varyani F, Ostergren J, Michelson EL, Young JB (2008). Impact of diabetes on outcomes in patients with low and preserved ejection fraction heart failure: an analysis of the Candesartan in Heart failure: assessment of Reduction in Mortality and morbidity (CHARM) programme. Eur Heart J.

[3] Kishi S, Gidding SS, Reis JP, Colangelo LA, Venkatesh BA, Armstrong AC (2017). Association of insulin resistance and glycemic metabolic abnormalities with LV structure and function in middle age: the cardia study. JACC Cardiovasc Imaging..

[4] Zhang X, Wei X, Liang Y, Liu M, Li C, Tang H (2013). Differential changes of left ventricular myocardial deformation in diabetic patients with controlled and uncontrolled blood glucose: a three-dimensional speckle-tracking echocardiography-based study. J Am Soc Echocardiogr.

[5] Tang X, Li S, Wang Y, Wang M, Yin Q, Mu P (2016). Glycemic variability evaluated by continuous glucose monitoring system is associated with the 10-y cardiovascular risk of diabetic patients with well-controlled HbA1c. Clin Chim Acta.

[6] Lin CC, Yang CP, Li CI, Liu CS, Chen CC, Lin WY (2014). Visit-to-visit variability of fasting plasma glucose as predictor of ischemic stroke: competing risk analysis in a national cohort of Taiwan Diabetes Study. BMC Med..

[7] Muggeo M, Verlato G, Bonora E, Zoppini G, Corbellini M, de Marco R (1997). Long-term instability of fasting plasma glucose, a novel predictor of cardiovascular mortality in elderly patients with non-insulin-dependent diabetes mellitus: the Verona Diabetes Study. Circulation.

[8] Lee CL, Sheu WH, Lee IT, Lin SY, Liang WM, Wang JS (2018). Trajectories of fasting plasma glucose variability and mortality in type 2 diabetes. Diabetes Metab.

[9] Xu D, Fang H, Xu W, Yan Y, Liu Y, Yao B (2016). Fasting plasma glucose variability and all-cause mortality among type 2 diabetes patients: a dynamic cohort study in Shanghai, China. Sci Rep.

[10] Hirakawa Y, Arima H, Zoungas S, Ninomiya T, Cooper M, Hamet P (2014). Impact of visit-to-visit glycemic variability on the risks of macrovascular and microvascular events and all-cause mortality in type 2 diabetes: the ADVANCE trial. Diabetes Care.

[11] Muggeo M, Zoppini G, Bonora E, Brun E, Bonadonna RC, Moghetti P (2000). Fasting plasma glucose variability predicts 10-year survival of type 2 diabetic patients: the Verona Diabetes Study. Diabetes Care.

[12] Alberti KG, Zimmet PZ (1998). Definition, diagnosis and classification of diabetes mellitus and its complications. Part 1: diagnosis and classification of diabetes mellitus provisional report of a WHO consultation. Diabetic Med..

[13] Williams B, Mancia G, Spiering W, Agabiti Rosei E, Azizi M, Burnier M (2018). 2018 Practice Guidelines for the management of arterial hypertension of the European Society of Hypertension and the European Society of Cardiology: ESH/ESC Task Force for the Management of Arterial Hypertension. J Hypertens.

[14] Catapano AL, Graham I, De Backer G, Wiklund O, Chapman MJ, Drexel H (2017). 2016 ESC/EAS guidelines for the management of dyslipidaemias. Rev Esp Cardiol (Engl Ed)..

[15] Matthews DR, Hosker JP, Rudenski AS, Naylor BA, Treacher DF, Turner RC (1985). Homeostasis model assessment: insulin resistance and beta-cell function from fasting plasma glucose and insulin concentrations in man. Diabetologia.

[16] Kilpatrick ES, Rigby AS, Atkin SL (2008). A1C variability and the risk of microvascular complications in type 1 diabetes: data from the Diabetes Control and Complications Trial. Diabetes Care.

[17] Lang RM, Bierig M, Devereux RB, Flachskampf FA, Foster E, Pellikka PA (2005). Recommendations for chamber quantification: a report from the American Society of Echocardiography’s Guidelines and Standards Committee and the Chamber Quantification Writing Group, developed in conjunction with the European Association of Echocardiography, a branch of the European Society of Cardiology. J Am Soc Echocardiogr.

[18] Davies MJ, D’Alessio DA, Fradkin J, Kernan WN, Mathieu C, Mingrone G (2018). Management of Hyperglycemia in Type 2 Diabetes, 2018 A Consensus Report by the American Diabetes Association (ADA) and the European Association for the Study of Diabetes (EASD). Diabetes Care..

[19] Yoshida M, Mita T, Yamamoto R, Shimizu T, Ikeda F, Ohmura C (2012). Combination of the Framingham risk score and carotid intima-media thickness improves the prediction of cardiovascular events in patients with type 2 diabetes. Diabetes Care.

[20] Fang ZY, Schull-Meade R, Downey M, Prins J, Marwick TH (2005). Determinants of subclinical diabetic heart disease. Diabetologia.

[21] Fang ZY, Leano R, Marwick TH (2004). Relationship between longitudinal and radial contractility in subclinical diabetic heart disease. Clin Sci (London, England : 1979).

[22] Maiello M, Zito A, Cecere A, Ciccone MM, Palmiero P (2017). Left ventricular diastolic dysfunction in normotensive postmenopausal women with type 2 diabetes mellitus. Cardiol J..

[23] Mochizuki Y, Tanaka H, Matsumoto K, Sano H, Toki H, Shimoura H (2015). Clinical features of subclinical left ventricular systolic dysfunction in patients with diabetes mellitus. Cardiovasc Diabetol.

[24] Enomoto M, Ishizu T, Seo Y, Yamamoto M, Suzuki H, Shimano H (2015). Subendocardial systolic dysfunction in asymptomatic normotensive diabetic patients. Circ J.

[25] Poulsen MK, Henriksen JE, Dahl J, Johansen A, Gerke O, Vach W (2010). Left ventricular diastolic function in type 2 diabetes mellitus: prevalence and association with myocardial and vascular disease. Circ Cardiovasc Imaging.

[26] Kozakova M, Morizzo C, Fraser AG, Palombo C (2017). Impact of glycemic control on aortic stiffness, left ventricular mass and diastolic longitudinal function in type 2 diabetes mellitus. Cardiovasc Diabetol.

[27] Zoppini G, Bonapace S, Bergamini C, Rossi A, Trombetta M, Lanzoni L (2016). Evidence of left atrial remodeling and left ventricular diastolic dysfunction in type 2 diabetes mellitus with preserved systolic function. Nutr Metab Cardiovasc Dis.

[28] Wang Y, Hou D, Ma R, Ding G, Yin L, Zhang M (2016). Early detection of left atrial energy loss and mechanics abnormalities in diabetic patients with normal left atrial size: a study combining vector flow mapping and tissue tracking echocardiography. Med Sci Monit.

[29] Ceyhan K, Kadi H, Koc F, Celik A, Ozturk A, Onalan O (2012). Longitudinal left ventricular function in normotensive prediabetics: a tissue Doppler and strain/strain rate echocardiography study. J Am Soc Echocardiogr.

[30] Ernande L, Bergerot C, Rietzschel ER, Buyzere ML, Thibault H, Pignonblanc PG (2011). Diastolic dysfunction in patients with type 2 diabetes mellitus: is it really the first marker of diabetic cardiomyopathy?. J Am Soc Echocardiogr..

[31] Vinereanu D, Nicolaides E, Tweddel AC, Madler CF, Holst B, Boden LE (2003). Subclinical left ventricular dysfunction in asymptomatic patients with Type II diabetes mellitus, related to serum lipids and glycated haemoglobin. Clin Sci..

[32] Cardoso CRL, Leite NC, Moram CBM, Salles GF (2018). Long-term visit-to-visit glycemic variability as predictor of micro- and macrovascular complications in patients with type 2 diabetes: the Rio de Janeiro Type 2 Diabetes Cohort Study. Cardiovasc Diabetol.

[33] Matsutani D, Sakamoto M, Minato S, Kayama Y, Takeda N, Horiuchi R (2018). Visit-to-visit HbA1c variability is inversely related to baroreflex sensitivity independently of HbA1c value in type 2 diabetes. Cardiovasc Diabetol.

[34] Gu J, Pan JA, Fan YQ, Zhang HL, Zhang JF, Wang CQ (2018). Prognostic impact of HbA1c variability on long-term outcomes in patients with heart failure and type 2 diabetes mellitus. Cardiovasc Diabetol.

[35] Wu W, Sun Z, Li Q, Wang M, Miao J, Zheng Z (2012). Influence of the glucose-lowering rate on left ventricular function in patients with type 2 diabetes and coronary heart disease. J Diabetes Complications.

[36] Takahashi H, Iwahashi N, Kirigaya J, Kataoka S, Minamimoto Y, Gohbara M (2018). Glycemic variability determined with a continuous glucose monitoring system can predict prognosis after acute coronary syndrome. Cardiovasc Diabetol.

[37] Fuentes-Antras J, Picatoste B, Ramirez E, Egido J, Tunon J, Lorenzo O (2015). Targeting metabolic disturbance in the diabetic heart. Cardiovasc Diabetol.

[38] Fontes-Carvalho R, Ladeiras-Lopes R, Bettencourt P, Leite-Moreira A, Azevedo A (2015). Diastolic dysfunction in the diabetic continuum: association with insulin resistance, metabolic syndrome and type 2 diabetes. Cardiovasc Diabetol.

[39] Bugger H, Abel ED (2014). Molecular mechanisms of diabetic cardiomyopathy. Diabetologia.

[40] Seferovic PM, Paulus WJ (2015). Clinical diabetic cardiomyopathy: a two-faced disease with restrictive and dilated phenotypes. Eur Heart J..

[41] From AM, Scott CG, Chen HH (2009). Changes in diastolic dysfunction in diabetes mellitus over time. Am J Cardiol.

[42] Park J, Kim JS, Kim SH, Kim S, Lim SY, Lim HE (2017). Subclinical left ventricular diastolic dysfunction and incident type 2 diabetes risk: the Korean Genome and Epidemiology Study. Cardiovasc Diabetol.

[43] Reis JP, Allen NB, Bancks MP, Carr JJ, Lewis CE, Lima JA (2018). Duration of diabetes and prediabetes during adulthood and subclinical atherosclerosis and cardiac dysfunction in middle age: the CARDIA study. Diabetes Care.

[44] Bibra H, Siegmund T, Ceriello A, Volozhyna M, Schumm-Draeger PM (2009). Optimized postprandial glucose control is associated with improved cardiac/vascular function—comparison of three insulin regimens in well-controlled type 2 diabetes. Horm Metab Res.

[45] Monnier L, Mas E, Ginet C, Michel F, Villon L, Cristol JP (2006). Activation of oxidative stress by acute glucose fluctuations compared with sustained chronic hyperglycemia in patients with type 2 diabetes. JAMA.

[46] Nishikawa T, Edelstein D, Du XL, Yamagishi S, Matsumura T, Kaneda Y (2000). Normalizing mitochondrial superoxide production blocks three pathways of hyperglycaemic damage. Nature.

[47] Esposito K, Nappo F, Marfella R, Giugliano G, Giugliano F, Ciotola M (2002). Inflammatory cytokine concentrations are acutely increased by hyperglycemia in humans: role of oxidative stress. Circulation.

[48] Paulus WJ, Tschope C (2013). A novel paradigm for heart failure with preserved ejection fraction: comorbidities drive myocardial dysfunction and remodeling through coronary microvascular endothelial inflammation. J Am Coll Cardiol.

[49] Crespo MJ, Zalacain J, Dunbar DC, Cruz N, Arocho L (2008). Cardiac oxidative stress is elevated at the onset of dilated cardiomyopathy in streptozotocin-diabetic rats. J Cardiovasc Pharmacol Ther.

[50] Kajstura J, Fiordaliso F, Andreoli AM, Li B, Chimenti S, Medow MS (2001). IGF-1 overexpression inhibits the development of diabetic cardiomyopathy and angiotensin II-mediated oxidative stress. Diabetes.

[51] Baynes JW (1991). Role of oxidative stress in development of complications in diabetes. Diabetes.

[52] Ying C, Liu T, Ling H, Cheng M, Zhou X, Wang S (2017). Glucose variability aggravates cardiac fibrosis by altering AKT signalling path. Diabetes Vasc Dis Res..

[53] Magri CJ, Mintoff D, Camilleri L, Xuereb RG, Galea J, Fava S (2018). Relationship of hyperglycaemia, hypoglycaemia, and glucose variability to atherosclerotic disease in type 2 diabetes. J Diabetes Res.

[54] Kim NH, Kim DL, Kim KJ, Kim NH, Choi KM, Baik SH (2017). Effects of vildagliptin or pioglitazone on glycemic variability and oxidative stress in patients with type 2 diabetes inadequately controlled with metformin monotherapy: a 16-week, randomised, open label, Pilot Study. Endocrinol Metab (Seoul).

[55] Yamada H, Tanaka A, Kusunose K, Amano R, Matsuhisa M, Daida H (2017). Effect of sitagliptin on the echocardiographic parameters of left ventricular diastolic function in patients with type 2 diabetes: a subgroup analysis of the PROLOGUE study. Cardiovasc Diabetol.

