# Supplementary material for: Visit-to-visit fasting plasma glucose variability is an important risk factor for long-term changes in left cardiac structure and function in patients with type 2 diabetes
Source: Cardiovasc Diabetol. 2019 Apr 16;18:50. doi: 10.1186/s12933-019-0854-9 (PMC6469221; doi:10.1186/s12933-019-0854-9)
Supplement: Supplementary file 1 — Additional file 1: Table S1. Baseline and follow-up cardiac structural and functional parameters in participants. [file 12933_2019_854_MOESM1_ESM.doc]

**Additional table S1 Baseline and follow-up cardiac structural and functional parameters in participants**

| **Variables (% or mean ± SD)** | **Baseline** | **After** | ***P*-value** |
| --- | --- | --- | --- |
| **Left cardiac structure** |  |  |  |
| Left atrium, mm | 31.47 ± 5.26 | 33.53 ± 5.15 | <0.001 |
| Left ventricle, diastole, mm | 45.34 ± 4.39 | 48.42 ± 4.38 | <0.001 |
| Interventricular septum, mm | 12.64 ± 1.93 | 12.49 ± 2.02 | 0.261 |
| Left ventricular posterior wall thickness, diastole, mm | 9.46 ± 1.66 | 10.08 ± 1.70 | <0.001 |
| LV mass index, g/m^2^ | 101.80 ± 27.37 | 116.81 ± 32.75 | <0.001 |
| **Left ventricular systolic function** |  |  |  |
| Left ventricular ejection fraction, % | 58.00 ± 5.56 | 54.85 ± 7.00 | <0.001 |
| **Left ventricular diastolic function** |  |  |  |
| E velocity, cm/s | 0.77 ± 0.22 | 0.81 ± 0.22 | 0.012 |
| A velocity, cm/s | 0.84 ± 0.25 | 0.93 ± 0.27 | <0.001 |
| E/A ratio | 0.97 ± 0.34 | 0.92 ± 0.31 | 0.010 |
| Tissue Doppler e', cm/s | 0.11 ± 0.03 | 0.09 ± 0.03 | <0.001 |
| E/e' ratio | 7.66± 3.24 | 9.34 ± 3.84 | <0.001 |
